# Consumer Visual and Affective Bias for Soothing Dolls

**DOI:** 10.3390/ijerph20032396

**Published:** 2023-01-29

**Authors:** Yu-Hsiu Chu, Li-Wei Chou, He-Hui Lin, Kang-Ming Chang

**Affiliations:** 1 Department of Physical Therapy, Graduate Institute of Rehabilitation Science, China Medical University, Taichung 406040, Taiwan; 2 Department of Physical Medicine and Rehabilitation, Asia University Hospital, Asia University, Taichung 413505, Taiwan; 3 Department of Physical Medicine and Rehabilitation, China Medical University Hospital, Taichung 404332, Taiwan; 4 Department of Digital Media Design, Asia University, Taichung 413505, Taiwan; 5 Department of Computer Science and Information Engineering, Asia University, Taichung 413505, Taiwan; 6 Department of Medical Research, China Medical University Hospital, China Medical University, Taichung 406040, Taiwan

**Keywords:** visual preference, affective preference, International Affective Picture System, soothing dolls, eye tracker, self-assessment manikin

## Abstract

Soothing dolls are becoming increasingly popular in a society with a lot of physical and mental stress. Many products are also combined with soothing dolls to stimulate consumers’ desire for impulse buying. However, there is no research on the relationship between consumers’ purchasing behavior, consumers’ preference for soothing dolls, and visual preference. The purpose of this study was to examine the possible factors that affect the emotional and visual preferences of soothing dolls. Two local stores’ sales lists were used to extract three different types of dolls. The 2D and 3D versions of these three dolls were used. Subjective emotional preferences were examined by the self-assessment manikin (SAM) scale, with 5-point Likert scales for valence and arousal factors. An eye tracker was used to examine visual preferences, both before and after positive/negative emotion stimulation by the International Affective Picture System (IAPS). There were 37 subjects involved, with an age range of 20–28 years. The experimental results show that the average valence/arousal scores for 2D/3D dolls were (3.80, 3.74) and (2.65, 2.68), respectively. There was no statistical difference, but both 2D and 3D pictures had high valence scores. Eye tracker analysis revealed no gaze difference in visual preference between 2D and 3D dolls. After negative emotional picture stimulation, the observation time of the left-side doll decreased from 2.307 (std 0.905) to 1.947 (std 1.038) seconds, *p* < 0.001; and that of the right-side picture increased from 1.898 (std 0.907) to 2.252 (std 1.046) seconds, *p* < 0.001. The average observation time ratio of the eye on the 3D doll was 40.6%, higher than that on the 2D doll (34.3%, *p* = 0.02). Soothing dolls may be beneficial for emotion relaxation. Soothing dolls always have high valence features according to the SAM evaluation’s measurement. Moreover, this study proposes a novel research model using an eye-tracker and the SAM for the SOR framework.

## 1. Introduction

The pressure of life in modern society is very high, which also places pressure relief and healing in great demand. Many animated cute characters have been created and have become favorites with the spread of animation. These cute characters can not only make young children feel joy [1], but also can be used to treat sad and stressed patients [2,3]. Therefore, these cute characters are also called soothing dolls. Many types of merchandise are also matched with these soothing dolls to increase sales. Therefore, the healing business opportunity has become a popular area for sales. Consumers love soothing dolls and will involuntarily buy products with soothing doll labels; this purchasing action is related to impulse buying [4,5]. Impulse buying often occurs with online shopping [6]. Social networks, such as Facebook and Instagram, can have a significant impact on impulse buying [7]. Emotions of happiness and excitement both have positive effects on online impulse buying. The most widely studied affective stimuli include arousal, pleasure, positive emotions, and negative emotions [8]. The personality traits of online shoppers are slightly biased towards psychoticism [9], which also suggests that soothing dolls are helpful for emotional regulation, thus promoting purchasing behavior.

The classical purchasing behavior theory is Stimuli–Organism–Response (S-O-R), which examines the consumer’s purchasing behavior that is stimulated by products. In the S-O-R model, S is the stimulus, O is the consumer’s evaluation, and R is the response behavior [10]. Peng and Kim in 2014 used the S-O-R framework to study online shopping behavior. They examined how stimuli (consumers’ reasons for shopping and website stimuli) affected a consumer’s response (attitudes, adjustment to online shopping, ability to make emotional purchases, and repurchase intent) [11]. Zhu et al. studied the relationship between stimuli (perceived information quality and social presence) and response (purchase intention) generated by online reviews in 2020, and they found that the perceived information quality of online reviews and social presence had a positive impact on trust [12]. Another study showed that there was a close relationship between arousal–hedonic and pleasure–hedonic for consumer sentiment [13]. Due to COVID-19, the combination of online and offline business is a new trend in the post-COVID-19 era [14]. The use of highly emotional stimulation to increase consumption is an interesting research topic. In addition, the scope of neuromarketing is to study a subject’s feelings and reflections under highly emotional stimuli [15].

Neuromarketing evaluates consumers’ preferences and behavior using physiological measurements [16]. These physiological measurements include eye trackers, brain waves, heartbeat signals, muscle contractions, and body motions [17]. Among these measurements, the eye tracker is one of the most used tools [18]. The eye tracker can monitor the gaze position and observation length of consumers on the product. Although high visual preference may not be associated with shopping decisions [19], the eye tracker is still a popular tool for the study of online shopping behavior. Boardman and McCormick applied eye trackers to the study of consumer attention, and cognitive and emotional responses, for online fashion retail websites. They found that consumer attention and behavior vary throughout the shopping journey based on web content, functionality, and consumer goals. Top-down attention is more dominant than bottom-up attention. The most visited and time-consuming page of a shopping website is the product listing page [20]. Fei et al. investigated social cues in e-commerce based on the S-O-R model and an eye tracker. Their results showed that both herding messages and interaction text can attract exogenous attention. Anchor characteristics (i.e., attractiveness) may moderate the impact of social cues. The role of exogenous attention is complex, involving the competing mechanisms of a distracting effect and social influence [21]. Therefore, the eye tracker has a significant effect in the research of consumers’ attention patterns.

In addition to instruments, questionnaires are also a very important tool for investigating consumer’s preferences. The self-assessment manikin (SAM) is a commonly used emotion assessment questionnaire. The SAM is a way of assessing emotional experience through subjective ranking of pictures. The SAM ranking emotions include valence, arousal, and dominance [22,23]. Combined with the S-O-R model, Sheng and Joginapelly found consumers’ emotional response plays an important role in predicting and measuring behavioral intentions and satisfaction in online purchases. The S-O-R model was used to examine the influence of online atmosphere cues (mainly vividness and interactivity) on users’ emotional responses in e-commerce and the influence of users’ emotional responses on their purchase intentions. Users with higher arousal and positive valence reported increased intention to buy from e-commerce sites [24]. The combination of S-O-R and SAM has also been used to investigate the relationship between the destination physical servicescape elements, perceived price, tourist emotions, satisfaction, and behavioral intentions [25]. Consumers’ viewing emotions are also factors that affect consumer behavior. In operational experiments on subjects’ emotions, the most common practice is to ask subjects to look at a series of pictures in advance. The sentiment indicators of these pictures must be clearly defined. The International Affective Picture System (IAPS) is a group of recognized emotional pictures. These pictures contain three dimensions: valence, arousal, and dominance [26]. For each IAPS picture, there is a corresponding SAM ranking based on an evaluation of a huge number of subjects. Each IASP picture can correspond to an emotion based on the Russel emotion model [27]. By viewing a group of IAPS pictures with similar emotions, the subjects can be made to have similar emotions. For example, the subjects were asked to watch a series of high valence IAPS pictures in the study of the relationship between high valence emotion and the bidding behavior in online auctions [28]. IAPS also can be used as a neutral emotion standard compared with the emotional preference of shopping pictures to estimate the shopping behavior of users with online-buying-shopping disorder [29]. Therefore, IAPS is beneficial for emotion regulation with the aid of SAM ranking.

The goal of this study was to explore consumers’ visual and emotional preferences for soothing dolls. Two- and three-dimensional pictures of soothing dolls were compared. In addition, the consumer’s emotion was also regulated by an IAPS high valence and low valence picture set. The visual preference was examined by an eye tracker, and the subjective emotion preference was examined using a SAM ranking. Although there was no corresponding purchasing behavior of the subjects, market sales data were included as the soothing doll selection platform. The following hypotheses were developed for this study:

**H1.**  *There is a visual preference difference between 2D and 3D soothing doll pictures.*

**H2.**  *There is a subjective emotion preference difference between 2D and 3D soothing doll pictures.*

**H3.**  *There is a subjective emotion preference difference among the three different types of soothing dolls, either in 2D or 3D.*

**H4.**  *There is a visual preference difference after emotional manipulation by IAPS.*

**H5.**  *There is a visual preference difference between two dolls on the right side and on the left side.*

**H6.**  *There is a specific visual preference region on the soothing doll.*

**Figure 1:**
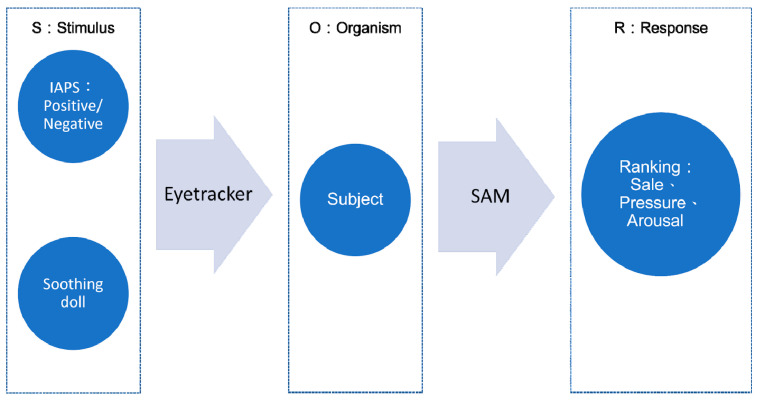
The modified S-O-R model for this study.

The modified S-O-R model for this study is demonstrated in Figure 1.

## 2. Methods

### 2.1. Subject Information

The recruitment conditions for this study were that participants were over 20 years of age, with vision correction of over 0.8, were male or female, and had normal sleep. A total of 37 subjects were recruited, aged 20 to 28. The mean age was 23.55 ± 2.26. The subjects were all familiar with the experimental procedure in advance and also filled out the consent form. They were required to complete the eye tracker measurement and SAM ranking for soothing dolls. The study was reviewed by the Research Ethics Committee of China Medical University Hospital in February 2022 (IRB number CRREC-110-133).

### 2.2. Soothing Doll Sales Data Collection

Soothing doll sales data were selected from two large local stores in Taiwan. These data are the daily sales ranking data published on the store’s website. The data collection period was from 2 March 2022 to 2 April 2022. The size range of the dolls in this study was defined as: no hanging, no pillow, and 15 to 60 cm on either side. Hanging dolls and large pillows were not within the scope of this study. During the data sampling period, the top ten sales rankings of each of the two stores were collected every day, and the screenshots were backed up and recorded. Next, a count was made of the number of times each doll that fits the size range of this study appeared in the top ten sales rankings, and of the ranking of each doll over 30 days. If both were included in the ranking, the maximum counting number reached 60. The sales data are shown in Table 1. The Sumikko gurashi ranked first, with a total counting number of 40.

### 2.3. Soothing Dolls Selection

From the results of Table 1, three dolls were chosen, that is, Sumikko gurashi, ranking first; NICI Pig Webby, ranking second; and Pui Pui Molcar, ranking fifth. The dolls ranked third and fourth were excluded due to having a similar shape as that of NICI Pig Webby. Both 2D and 3D picture website links of the three chosen dolls were collected for further SAM ranking and eye tracker experiments. The features of the three dolls are:

(Doll-1) Pui Pui Molcar: A face with obvious facial features. Labeled as P1-2 and P1-3, respectively, for 2D and 3D versions of the Pui Pui Molcar picture;

(Doll-2) Sumikko gurashi: A face without obvious facial features. Labeled as P2-2 and P2-3, respectively, for 2D and 3D versions of Sumikko gurashi;

(Doll-3) NICI Pig Webby: With distinct facial features and body. Labeled as P3-2 and P3-3, respectively, for 2D and 3D versions of NICI Pig Webby.

We also defined P1 = P1-2 + P1-3, P2 = P2-2 + P2-3, and P3 = P3-2 + P3-3.

### 2.4. Emotion Stimulation Pictures

The 12 selected IAPS pictures with high positive emotions were: 1440, 1463, 1710, 2040, 2154, 2340, 2347, 2550, 4574, 5833, 7200, and 8510. The valence scores of these pictures had an average of 7.80 (std 1.45), and arousal score average of 4.90 (std 2.42). These meet the requirements of high positive emotion. The other 12 pictures with negative emotions were: 2095, 2205, 2375.1, 2751, 2799, 2900.1, 3180, 9040, 9185, 9295, 9560, and 9830. The valence scores of these pictures averaged 2.18 (std 1.43), and the arousal scores averaged 5.18 (std 2.26). These meet the requirements of negative emotion, with a 9-point Likert scale. Each selected IAPS picture description and corresponding valence/arousal score are listed in Appendix A.

### 2.5. SAM Ranking of Soothing Dolls

In this study, the valence and arousal of the SAM ranking were used. This study used a 5-point Likert scale, with one point representing low pleasure or low arousal, and five points representing high pleasure or high arousal. The valence/arousal item refers to the degree of valence/arousal that this picture evokes in the subject. These two questions were asked separately and were answered while seeing all the pictures at the same time. The illustration of the SAM is shown in Appendix B. Due to COVID-19, this study adopted online answering. The six pictures and scoring questions were placed in Excel, and the subjects completed the answers under the online voice guidance of the researcher.

### 2.6. Eyetracker Recording

**Figure 2:**
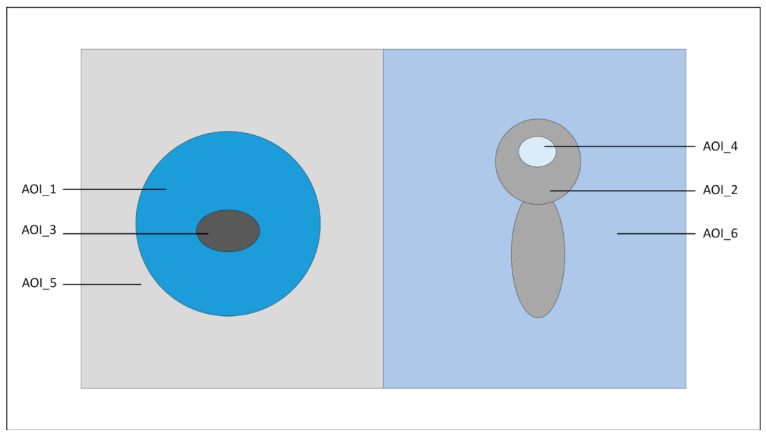
Eye tracker soothing dolls’ position and AOI.

The Eyetech eye tracking system (VT2 Mini, Mangold Vision, sampling frequency 60 Hz) was used in this experiment. Before eye tracker recording, calibration was conducted for each subject to ensure that the gaze position was the same as the picture position. Six pictures were used for the SAM ranking and eye tracker, inclusive of the three soothing dolls, where each doll had one 2D and one 3D picture. Two randomly chosen pictures were placed on the left and right screen, as shown in Figure 2. There were a total of 30 arrangements. In this way, doll pictures appeared in pairs in different groups, for example: P1-2 vs. P1-3, or P1-2 vs. P2-3. Each picture group was shown for 5 s. There were two stages. There were 15 picture groups shown in each stage. Eye tracker data were recorded at each stage. There was a 60 s break between the two stages. After the break, subjects were required to watch a set of IAPS-derived emotion pictures. There were two IAPS groups, positive and negative emotion groups. Each subject was randomly assigned to one of the groups. There were 12 IAPS pictures in each group. Each IAPS picture lasted for 10 s. The second stage followed another 30 s break. The total time of the eye tracker experiment for each subject was around 6 min.

### 2.7. Eye Tracker Feature Extraction

The area of interest (AOI) is illustrated in Figure 2 and was defined as follows:

AOI-1: left doll;

AOI-2: right doll;

AOI-3: eye of left doll;

AOI-4: eye of right doll;

AOI-5: ALL left half;

AOI-6: ALL right half;

Eye_ratio = AOI-3/AOI-1 + AOI-4/AOI-2.

The following eye tracker features were extracted: observation length, fixation count, and time to first fixation. The eye tracker heat map was also extracted.

**Figure 3:**
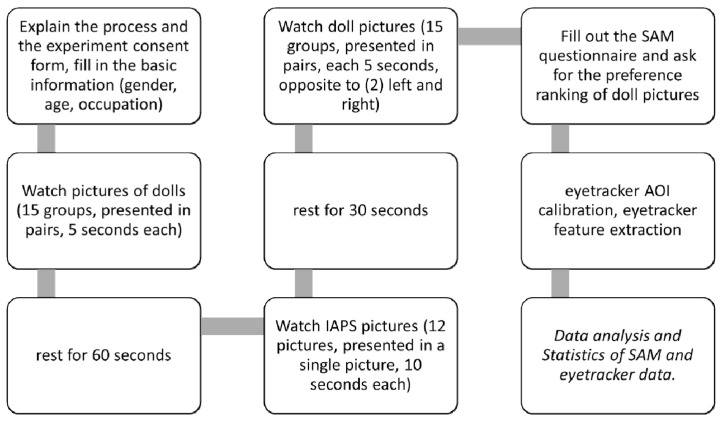
Experiment flowchart.

The experiment flowchart is shown in Figure 3.

### 2.8. Statistics

#### 2.8.1. Descriptive Statistics

The average and standard derivation of the following features were estimated:

SAM valence/arousal ranking of 6 doll pictures.

Three eye tracker features: observation length, fixation count, and time to first fixation, derived from 2D/3D, and derived from IAPS positive/negative stimulation groups for the first and second stages.

#### 2.8.2. *t*-Test

The following feature differences were examined using a *t*-test for three eye tracker features:

2D vs. 3D at both stage 1 and stage 2;

AOI-5 vs. AOI-6;

Stage 1 vs. stage 2 at both IAPS positive/negative groups.

#### 2.8.3. Chi Square Test

The differences in the SAM ranking for the six doll pictures were examined by the chi square test. Both valence and arousal ranking differences were estimated for the following differences:

Pi-2 vs. Pi-3, i = 1,2,3;

P1 vs. P2;

P1 vs. P3;

P2 vs. P3;

P1 vs. P2 vs. P3.

All data were organized and analyzed using Google Sheet software.

## 3. Results

The SAM distribution of the six soothing doll pictures is listed in Table 2. The SAM is a 5-point Likert scale survey. The valence ranged from 3.65 to 4.03, and arousal ranged from 2.46 to 2.89. The average valence was high for all six soothing doll pictures.

The SAM distribution difference of the six soothing doll pictures estimated by the chi square test is listed in Table 3. There was no difference in the valence among P1, P2, and P3; here, the only difference was for 2D versus 3D on P3 of the arousal ranking (*p* = 0.047). Overall, these six pictures resulted in almost no difference and had a high valence ranking.

The eye tracker feature distribution of 2D and 3D soothing dolls before and after IAPS emotion stimulation is shown in Table 4. There was no difference among 2D and 3D doll pictures for eye tracker features, either in stage 1 or stage 2.

The eye tracker feature distribution of right-side and left-side soothing doll pictures with positive/negative IAPS emotion stimulation is shown in Table 5. There was no difference for the positive emotion stimulus group, but there was significant difference for the negative emotion stimulus group. For the negative group, the main observation moved from left-side pictures to right-side pictures after negative emotion stimulation.

**Figure 4:**
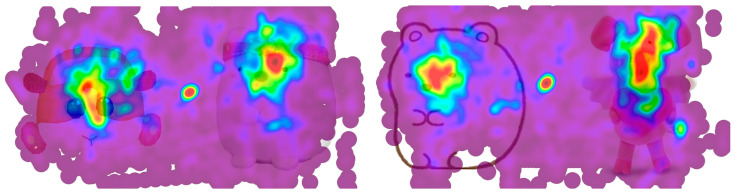
Illustration of the eye tracker heat map. The data are the combination of all subject eye tracker observation lengths for the same doll picture. Left: P1-2 vs. P2-3; Right: P2-2 vs. P3-3.

The typical heat maps of three dolls are demonstrated in Figure 4. From Figure 4, it can be found that the eye area is the main focus area of the three dolls, and the other peripheral areas of the dolls receive less attention. The center point of the picture has an additional red focus, which may be the result of the movement of the eyes of the two dolls, which do not attract the subjects.

The eye tracker feature distribution of the eye region on soothing doll pictures is shown in Table 6. There were significant differences between 2D and 3D pictures, either on stage 1 or stage 2, for both observation length and observation count features. Compared with the whole doll, the observation duration ratio of 3D was also higher than the 2D ratio.

According to the result shown above, three of the proposed hypotheses are valid, whereas the other three are not valid, as shown in Table 7.

## 4. Discussion

There are some interesting findings of this study. The soothing dolls were selected according to the sales ranking. The shapes of these three dolls differ. Only one doll has a shape inclusive of the head and body; the other two dolls only have heads. The 2D and 3D versions were also included. According to Hypotheses 1 to 3, there was no visual preference difference between 2D and 3D soothing doll pictures, based on the SAM ranking and eye tacker. Although there was no difference between SAM 2D and 3D, the valence rankings of the three dolls were very high, which is in line with the characteristics of soothing and the sales ranking. This finding can be a useful guide for future design of new soothing dolls. The SAM ranking of newly designed dolls must have a high valence.

Another interesting finding is that Hypotheses H4 and H5 are valid. After watching IAPS emotion sets, subjects may feel happier or sadder. The negative IAPS emotion set groups try to simulate someone in a bad mood, and to determine how soothing dolls work for soothing. From the eye tracker data, there were differences on the left- and right-side dolls between stage 1 and stage 2 for the negative IAPS emotion groups. The participants tended to focus on the right side after negative IASP stimulation. This is very interesting. The reason for this may be that the right hemisphere is more involved in responding to emotion than the left hemisphere [30,31]. When subjects focus on the right side, their left hemisphere works more than their right hemisphere. These subjects may be more rational and calmer. This finding indirectly suggests that the selected dolls are soothing and healing. However, the data presented so far are not enough to explain this matter, and other tools, such as EEG, are needed to evaluate this hypothesis in the future. Hypothesis H6 is valid. According to the eye tracker data, subjects spend more time on the eye region of the soothing dolls than other regions. The observation ratio of the eye on 3D is higher than that on 2D. Similar results were obtained in studies using eye trackers to observe pictures of real people, showing that the face and eyes are the main areas of the gaze [32].

This study also proposes a modified S-O-R model. In this study, the eye tracker was placed in the S-O mediation and the SAM was placed in the O-R mediation. However, these two tools are not just a simple mediation process, they may each cover a part of O. Figure 1 shows only a simplified model. The two arrows overlap with O, and we cannot know how much they are in O. It is very likely that both the eye tracker and the SAM can be placed in O to show the psychological activity of consumers. R is the subsequent shopping behavior. Behavior during the COVID-19 crisis appears to be consistent with behavior exhibited during historic shock events [33]. Under negative and highly emotional stimulation, in which everyone is nervous about the epidemic, impulsive shopping behavior is enhanced [5], which indirectly leads to the demand for soothing dolls. This was also one of the research motivations of this paper. Moreover, this study proposes a novel research model comprising the eye tracker and SAM for the S-O-R framework.

Although soothing dolls have become more widely available in recent years, little research exists to support the claims regarding their ability to improve mood. Dolls have historically been used in art therapy and emotion; nevertheless, the mechanism of doll treatment is unclear [3], and the study methodologies are largely limited to interviews or opinions [34]. To better understand customers’ preferences for comforting dolls, this study presents a novel approach employing the SAM and eye tracker to quantify emotional and visual preferences. Even though these two research methods are frequently used in neuromarketing, they are extremely recent and helpful methods for the study of comforting dolls.

There are several limitations of this study. The sales survey only included two stores. This may have missed other soothing dolls that were not shown in the store list. In addition, for the same doll, only one type was selected; thus, the number of dolls was relatively small. This is a major limitation of this pilot study. The purpose of this study was to investigate the applicability of visual preference and subjective emotion preference on soothing dolls. The follow-up research will include more dolls with which to compare. In addition, these dolls were taken from lists of items having high sales and that were also popular items. These dolls have many corresponding animations, movies, etc. Therefore, the emotional preference of watching these dolls will also carry the impression of the doll in the past, not just the emotional preference due to the doll’s shape. This study also only discussed visual and emotional preferences but did not discuss the impact of past tactile memory. There is also a relationship between touch and therapy, but the perception of touch was not discussed in this study. In addition, the data of the sales rankings are not the same subjects as those of the eye tracker experiment and the SAM evaluation, and there may be a certain degree of data error. However, this part is not easy to overcome, because there will be differences between market data and laboratory control experiments. Other papers treat the data in the same way [24].

## 5. Conclusions

The most popular soothing doll is Sumikko gurashi, according to the market sales ranking. According to the finding of the eye tracker, the gaze patterns are similar among the three dolls. The observation around the eye is higher for 3D dolls than that for 2D dolls. Subjects with negative IAPS emotion set stimulation tended to look at the pictures on the right, which may be related to the rational and emotional stability of the left brain. This also indirectly shows that soothing dolls have the effect of healing and emotional stabilization. These dolls, selected from those having high market sales, also have high valence and a moderately high arousal ranking according to the SAM ranking survey, so the SAM data can also be used as a standard for future soothing doll design.

Additionally, we used a cutting-edge concept known as neuromarketing for impulsive purchasing. To determine whether the impulsive buyer inclined to purchase a soothing doll had any unique visual or emotional preferences, we conducted the study using the SAM and eye tracker, both of which are frequently utilized in neuromarketing. This is a fascinating attempt because soothing dolls are essentially designed to provoke a preference and can affect emotions.

## Figures and Tables

**Table 1:** Soothing doll sales data distribution.

Ranking	Soothing Doll	Counting Number
1	Sumikko gurashi	40
2	NICI Pig Webby	30
3	Disney	20
4	Panda	19
5	Pui Pui Molcar	8
6	Penguin	7
7	Qiaohu	6
7	Shiba Inu	6
9	Little twins stars unicorn	5
10	Jinbesan	2
10	Jujutsu Kaisen	2

**Table 2:** SAM distribution of 6 soothing doll pictures.

	Valence		Arousal	
	Mean	std	Mean	std
P1-2	4.03	0.96	2.89	1.26
P1-3	3.65	1.16	2.70	1.29
P2-2	3.65	0.98	2.35	1.23
P2-3	3.78	0.89	2.46	1.12
P3-2	3.70	0.88	2.57	1.12
P3-3	3.81	1.05	2.78	1.46

**Table 3:** SAM chi square test.

	Valence	Arousal
P1-2 vs. P1-3	0.335	0.786
P2-2 vs. P2-3	0.780	0.474
P3-2 vs. P3-3	0.490	0.047
P1 vs. P2	0.335	0.317
P1 vs. P3	0.168	0.538
P2 vs. P3	0.849	0.568
P1 vs. P2 vs. P3	0.380	0.564

**Table 4:** Eye tracker result of 2D and 3D soothing dolls. (Data are derived from the sum of either AOI-1 or AOI-2).

			Observation Length (s)	Fixation Count	Time to First Fixation (s)
stage 1	2D	Mean	1.586	102.1	0.500
		Std	0.829	65.0	0.638
	3D	Mean	1.699	111.7	0.427
		Std	0.826	64.3	0.678
		*p* value	0.399	0.281	0.440
stage 2	2D	Mean	1.542	104.6	0.457
		Std	0.975	72.3	0.813
	3D	Mean	1.526	106.4	0.422
		Std	0.953	71.1	0.586
		*p* value	0.917	0.861	0.724

**Table 5:** Eye tracker result of IAPS stimulation and left–right position factors.

			Observation Length (s)-AOI5	Observation Length (s)-AOI6	*p* Value	Fixation Count-L	Fixation Count-R	*p*
Positive	stage 1	Mean	2.055	2.103	0.663	139.9	136.7	0.657
		Std	0.961	0.926		76.0	71.7	
	stage 2	Mean	2.005	2.178	0.140	138.1	150.0	0.163
		Std	0.988	1.007		80.0	84.2	
		*p* value	0.525	0.334		0.770	0.023	
Negative	stage 1	Mean	2.318	1.885	0.00010	139.361	117.115	0.00197
		Std	0.919	0.919		76.694	72.466	
	stage 2	Mean	1.972	2.225	0.04684	121.802	133.823	0.12833
		Std	1.052	1.058		78.509	81.077	
		*p* value	0.00008	0.00010		0.00440	0.01459	

**Table 6:** Eye tracker results of AOI around the eyes. The data were derived from AOI-3 and AOI-4.

			Observation Length (s) on Eye	Observation Length (s) on Eye Ratio	Fixation Count	Time to First Fixation (s)
Stage 1	2D	Mean	0.557	33.19%	37.748	0.864
		Std	0.543	0.248	40.707	1.092
	3D	Mean	0.713	39.43%	47.901	0.820
		Std	0.636	0.291	46.831	1.157
		*p* value	0.03174	0.03377	0.04750	0.74446
Stage 2	2D	Mean	0.474	25.89%	33.063	0.828
		Std	0.534	0.230	39.959	1.347
	3D	Mean	0.605	33.96%	42.712	0.671
		Std	0.584	0.246	43.096	0.973
		*p* value	0.07354	0.00099	0.06522	0.33378

**Table 7:** Hypothesis checking.

Hypothesis Order	Content	Valid or not
H1	There is a visual preference difference between 2D and 3D soothing doll pictures.	Not
H2	There is a subjective emotion preference difference between 2D and 3D soothing doll pictures.	Not
H3	There is a subjective emotion preference difference among the three body-type soothing dolls, either in 2D or 3D.	Not
H4	There is a visual preference difference after emotional manipulation by IAPS.	Valid
H5	There is a visual preference difference between two dolls on the right side and on the left side.	Valid
H6	There is a specific visual preference region on the soothing doll.	Valid

## Data Availability

Not applicable.

## Appendix A

The IAPS information used in this study is available at the following links: available online: https://figshare.com/s/b2f4b7866f78e140bfe8 (accessed on 12 December 2022).

## Appendix B

The SAM ranking used in this study is available at the following links: available online: https://figshare.com/s/c64b9014c8261540b62f (accessed on 12 December 2022).
